# Association between obstetric mode of delivery and emotional and behavioural problems in children and adolescents: the children of the 90s health study

**DOI:** 10.1007/s00127-022-02374-z

**Published:** 2022-10-14

**Authors:** Berihun Assefa Dachew, Gizachew A. Tessema, Rosa Alati

**Affiliations:** 1 School of Population Health, Curtin University, Perth, Australia; 2 School of Public Health, University of Adelaide, Adelaide, Australia; 3 Institute for Social Science Research, The University of Queensland, Brisbane, Australia

**Keywords:** ALSPAC, Behavioural problems, Emotional problems, Mode of delivery, Offspring

## Abstract

**Purpose:**

Existing evidence on the relationship between mode of delivery and offspring emotional and behavioural problems, especially in older age groups, is limited and inconsistent. This study aimed to examine the association between obstetric mode of delivery and emotional and behavioural problems in offspring aged 3–16 years.

**Methods:**

The sample for this study comprised participants in the Avon Longitudinal Study of Parents and Children (ALSPAC) in the United Kingdom. The study cohort ranged from 7074 (at 3 years of age) to 4071 (at 16 years of age) mother–offspring pairs. Data on obstetric mode of delivery were abstracted from obstetric records by trained research midwives and classified as spontaneous vaginal delivery, assisted vaginal delivery and caesarean delivery (elective and emergency). Offspring emotional and behavioural problems were measured using the Strengths and Difficulties Questionnaire (SDQ) when the child was 3, 7, 9, 11, and 16 years. Logistic regression analyses were used to examine associations.

**Results:**

Assisted vaginal delivery was associated with an increased risk of emotional problems at age 11 years (OR = 1.42; 95% CI 1.11–1.81). No significant associations were observed at ages 3, 7, 9 and 16. We found no evidence of associations between caesarean delivery (elective or emergency) and emotional and behavioural measures in offspring across all age groups.

**Conclusion:**

Mode of delivery does not appear to be associated with emotional and behavioural problems in children and adolescents. Further research is needed to understand the potential longer-term effects of assisted vaginal deliveries on offspring emotional development.

**Supplementary Information:**

The online version contains supplementary material available at 10.1007/s00127-022-02374-z.

## Introduction

The rates of caesarean section (CS) have been seen increasing worldwide, though to varying extents in different regions and countries [1]. Currently, CS accounts for 21% of births worldwide and is projected to be 30% by 2030 [2]. Most middle- and high-income countries have CS rates above the 10–15% of births [1, 2] which is thought to be optimal [3, 4]. For example, in the United Kingdom (UK), the rate of delivery CS was 26.2% in 2015 [1], more than twice the estimated 12.5% reported in 1990 [5]. Such high rates demand an understanding of the short- and long-term health consequences of caesarean delivery. On the other hand, the rate of assisted or operative vaginal delivery, which can be used to safely avoid some caesarean deliveries and thereby its complications, showed a decreasing trend [6]. Around 5.0% of all births in the United States were delivered by forceps in 1990, and this percentage decreased to 0.5% in 2019. A similar decline has been observed in the rate of vacuum delivery, from 5.9% in 1995 to 2.5% in 2019 [7].

Although CS is a life-saving intervention when medically indicated, there is no evidence showing the benefits of caesarean delivery for women or infants who do not require the procedure and may affect the health of the mother, her child, and future pregnancies [3, 4]. In light of this, several studies have reported the potential health risk of caesarean delivery in children, including altered immune development, increased risk of allergies and respiratory problems [8–10].

CS may affect behavioural development in children through altered gut microbiota, premature birth, and/or changes in maternal-child attachment [11–15]. Most babies get their first hefty dose of microbes during the birth process while travelling from a sterile environment in utero to the maternal birth canal [16, 17]. Children delivered by CS have been shown to have altered gut macrobiotic composition [14–17], and there is increasing evidence from both animal models and human subjects showing that the changes in the gut microbiota can alter brain physiology and behaviour [18–20]. A recent longitudinal study conducted in Australia found a strong association between the composition of the gut microbiota in infancy and behavioural problems at 2 years [18]. In another study, Christian et al. [21] also found associations between gut microbiome and maternal reported temperament in children aged 18–27 months. In animal model studies, it was found that rodents lacking all gut bacteria from birth developed behavioural abnormalities when compared to animals with normal gut microbiota [22, 23]. Although the mechanisms by which changes in the gut microbiota impact brain development and behaviour are not well studied, it appears to play an important role in stimulating neurodevelopment via neural, hormonal, and immunological signalling [18, 24].

Assisted vaginal delivery might also be associated with offspring emotional and behavioural development. One recognised complication of assisted vaginal delivery is the development of a subdural haematoma when a blood vessel in the space between the skull and the brain (the subdural space) is damaged [25]. Imaging studies showed that 60–67% of asymptomatic children born by vacuum or forceps delivery had subdural haemorrhage [26]. Such brain injury during delivery may increase the risk of mental disorders in later life. It is also important to note that fetal distress is a common reason for assisted vaginal delivery and is associated with adverse neurobehavioral development in children [27].

Existing evidence on the relationship between mode of delivery and offspring emotional and behavioural problems is limited and inconsistent. While some studies reported an increased risk of behavioural difficulties in offspring of mothers who gave birth via CS or assisted vaginal delivery [28–30], others reported no significant association [31–34]. Most of the existing studies examined the association at a one-time point only [28–32], did not distinguish between assisted vaginal delivery and spontaneous vaginal delivery and/or planned and emergency CS [28, 30, 33], did not examine specific emotional and behavioural outcomes (peer relationship behaviours, emotional problems, conduct problems, and hyperactivity/inattention problems) [28, 29, 31, 32], and have not consistently accounted for confounding factors, such as maternal depressive symptoms during pregnancy, parity, pre-eclampsia and gestational diabetes [28–30, 32], which are found to be linked to offspring behaviour problems. These limitations make it difficult to determine the true effect of obstetric mode of delivery. To address these limitations, this study aimed to examine the association between obstetric mode of delivery and emotional and behavioural problems in offspring at five developmental time points using a large longitudinal birth cohort study with the capacity to control for a range of known potential confounders. We hypothesised that children born via CS or assisted vaginal delivery would have more behavioural difficulties when compared with children born via spontaneous vaginal delivery.

## Methods

### Study participants

Our study sample comprised participants in the Avon Longitudinal Study of Parents and Children (ALSPAC) cohort, an ongoing population-based longitudinal birth cohort in Bristol, Avon, United Kingdom (UK). The core enrolled sample consisted of 14,541 pregnancies in women residents in the former county of Avon, the United Kingdom, who had an expected delivery date between 1st April 1991 and 31st December 1992 [35–37]. Of the 14,062 live births, 13,617 were singletons and alive at one year. In these analyses, we have included all women with singleton and live-born infants who had complete data on both the exposure (obstetric mode of delivery) and outcomes (child emotional and behavioural problem measures). 13,280 children had complete data on the obstetric mode of delivery at baseline. Data on the outcome variables were available for 9788 and 5502 children at the age of 3 and 16 years, respectively. The final analyses were conducted on children who had complete data on exposure, outcome and covariates, 7074 at age 3; 6114 at age 7; 5639 at age 9; 5211 at age 11; 4071 at age 16. Further details regarding recruitment, study design, and generalisability have been previously reported [35, 37]. The study website contains information on all the available data through a fully searchable data dictionary and variable search tool (http://www.bristol.ac.uk/alspac/researchers/our-data).

Ethical approval for the ALSPAC study was obtained from the ALSPAC Ethics and Law Committee as well as the Local Research Ethics Committees. Informed consent for the use of data collected via questionnaires and clinics was obtained from participants following the ALSPAC Ethics and Law Committee’s recommendations at the time. The details on the ethical approval, including the dates of approval and associated reference numbers, can be found in the ALSPAC Website [38].

### Outcome measures

Childhood emotional and behavioural problems were measured using the Strengths and Difficulties Questionnaire (SDQ), completed by parents, usually mothers, when the child was  3, 7, 9, 11, and 16 years [39]. The SDQ has previously been found to be a valid and reliable instrument used to screen emotional and behavioural problems in children aged 3–16 years and has been widely used in research and clinical practice [39, 40]. The tool encompasses 25 questions comprising five sub-scales: hyperactivity, emotional symptoms, conduct problems, peer problems, and pro-social behaviour (each comprising five items) [40]. Scores for the sub-scales range from 0 to 10, and the first four sub-scales are combined to calculate a total difficulties score, ranging from 0 to 40. A higher score indicates higher symptoms, except for pro-social behaviour, where a lower score indicates more difficulties [40]. Children were categorised as ‘borderline/abnormal’ if they scored in the ‘abnormal’ or ‘borderline ‘ranges, according to cut-offs suggested by Goodman [40] for total behavioural difficulties and for each SDQ sub-scale. Binomial cut-off: total difficulties (≥ 14), hyperactivity (≥ 6), conduct problems (≥ 3), emotional symptoms (≥ 4), peer- relationship problems (≥ 3), pro-social behaviour (≤ 5).

### Exposure measures

Data on obstetric mode of delivery were abstracted from obstetric records by trained research midwives. The mode of delivery was classified as spontaneous vaginal delivery, assisted vaginal delivery, and caesarean delivery (elective and emergency). Assisted vaginal delivery comprised assisted breech delivery, forceps delivery and vacuum extraction. Elective caesarean deliveries were those with prior planning, and emergency caesarean deliveries were those without prior planning or that took place after labour started.

### Confounding variables

Potential confounders were selected on the basis of previous reports of their association with obstetric mode of delivery and offspring emotional and behavioural problems [30–32, 41]. These include maternal age, education, marital status, ethnicity, parity, pregnancy diabetes status, hypertensive disorders during pregnancy, infection during pregnancy, maternal smoking and alcohol use during pregnancy, maternal pre-pregnancy body mass index (BMI), maternal antenatal depression and anxiety symptoms, offspring sex and gestational age at delivery. Data on these potential confounders were obtained from obstetric records and questionnaires administered during pregnancy.

Data on these potential confounders were obtained from obstetric records and questionnaires administered during pregnancy. Maternal education status was ascertained during pregnancy and split into five categories: Certificate of Secondary Education (CSE), vocational, O level (an examination taken and passed at 16 years of age), A level (examinations taken and passed at 18 years of age upon leaving secondary school), and university degree. Maternal marital status (never married, widowed/divorced/separated, and married) was obtained from questionnaires administered at baseline. Data on maternal age, gestational age at delivery, and maternal pregnancy diabetes status (no glycosuria or diabetes and existing diabetes, gestational diabetes or glycosuria) infection during pregnancy (yes, no) and parity (nullipara and multipara) were obtained from the obstetric records and questionnaires administered during pregnancy. Blood pressure and proteinuria were extracted from maternal obstetric records that were used to determine women with hypertensive disorders during pregnancy (yes/no). Self-reports of alcohol use were used to assess maternal alcohol use during pregnancy. Mothers were asked how often they had consumed alcoholic drinks during the first 3 months of pregnancy and dichotomised to yes and no. Mothers were dichotomised as smokers or non-smokers in response to self-reported smoking in the first 3 months of pregnancy. At the time of enrolment, mothers were asked to report their pre-pregnancy weight and height, which were used to calculate maternal pre-pregnancy BMI and classed as underweight (< 18.5 kg/m^2^), normal weight (18.5–24.9 kg/m^2^) and overweight/obese (≥ 25 kg/m^2^). Prenatal depression was measured at 32 weeks of gestation using Edinburgh Postnatal Depression Scale (EPDS), and scale scores were dichotomized using the recommended cut-off score for depression (12 out of 30)[42]. Symptoms of antenatal anxiety were measured with the Crown-Crisp Experiential Index (CCEI), and a score of 8 or more is used to indicate clinically significant anxiety symptoms [43].

### Statistical analysis

First, we described the sociodemographic and clinical characteristics of participants stratified by the mode of delivery using cross-tabulations and *χ*^2^ test statistics. Then, we conducted univariable and multivariable logistic regression to investigate the association between obstetric mode of delivery and emotional and behavioural problems in offspring across five measurement periods (i.e., 3, 7, 9, 11, and 16 years of age), computing ORs and 95% CIs as a measure of risk. The interaction between mode of delivery and gender was tested to account for gender differences in emotional and behavioural problems. Sensitivity analysis was performed by excluding preterm babies. To account for missing data, we conducted multiple imputations as a sensitivity analysis. We used 50 cycles of regression switching and generated 50 imputed data sets. All outcome data and covariates included in the regression model and additional auxiliary variables associated with incomplete variables were imputed, and the analyses were repeated. Statistical analyses were conducted using STATA software, release 16 [44]. All statistical tests were conducted with 2-tailed statistical significance levels set at *P* ≤ 0.05.

## Results

Table 1 shows the characteristics of study participants stratified by mode of delivery. Of 13,280 mothers who had data on mode of delivery, 10,175 (76.6%) had given birth by spontaneous vaginal delivery, 1542 (11.6%) by assisted delivery and 1397 (10.5%) by CS (3.7% elective CS and 6.8% emergency CS). The mean (SD) age of mothers who gave birth by spontaneous vaginal delivery was 27.6 (± 4.9). Compared with mothers who gave birth via spontaneous vaginal delivery, mothers who gave birth by CS were older at childbirth, educated, more likely to be nulliparous, overweight/obese, have hypertensive disorders during pregnancy and pregnancy diabetes, and less likely to smoke and drink alcohol during pregnancy. Mean (SD) birth weight of mothers who gave birth by spontaneous vaginal delivery was higher compared to those who gave birth by CS [3.4 (0.5) vs 3.2 (0.7), *P* < 0.001].

**Table 1:** Characteristics of study participants stratified by mode of delivery

Characteristics	Spontaneous vaginal delivery (*n* = 10175)	Assisted delivery (*n* = 1708)	Caesarean section (*n* = 1397)	*P* value
Maternal age at delivery (mean, SD)	27.6 (4.9)	27.6(4.7)	28.6(5.0)	< 0.001
Maternal education (*n*, %)				< 0.001
CSE	1351 (15.9)	144(9.9)	172(15.1)	
Vocational	940 (11.1)	121(8.3)	114(10.0)	
O level	3127 (36.8)	562(38.5)	406(35.7)	
A level	1955 (23.0)	389(26.6)	297(26.1)	
Degree	1120 (13.2)	244(16.7)	150(13.2)	
Marital status (*n*, %)				0.048
Married	7067 (74.4))	1199(75.7)	994(77.0)	
Widowed/divorced/separated	576 (6.1)	82(5.2)	85(6.6)	
Never married	1857 (19.6)	303(19.1)	212(16.4)	
Parity (*n*, %)				< 0.001
Nullipara	3561(37.9)	1279(81.6)	672(52.9)	
Multipara	5826(62.1)	288(18.4)	599(47.1)	
Pre-pregnancy BMI (Kg/m^2^) (*n*, %)				
< 18.5	436 (5.2)	73(5.1)	45(4.0)	< 0.001
18.5–24.99	6296 (75.0)	1086(75.5)	743(65.6)	
≥ 25	1666 (19.8)	280(19.4)	345(30.4)	
Hypertensive disorders during pregnancy (*n*, %)				
Yes	1386 (13.7)	375 (22.2)	393(28.6)	< 0.001
No	8725 (86.3)	1311(77.8)	981(71.4)	
Pregnancy diabetes status (*n*, %)				< 0.001
Glycosuria or diabetes (existing/gestational)	330 (3.6)	74 (4.8)	87 (7.0)	
No glycosuria or diabetes	8727 (96.4)	1465 (95.2)	1153(93.0)	
Any infection during pregnancy (*n*, %)				0.202
Yes	2020 (23.0)	310 (20.1)	280(23.4)	
No	6767 (77.0)	1168 (79.0)	918(76.6)	
Alcohol drinking in pregnancy (*n*, %)				0.027
Yes	5222 (55.2)	843 (53.5)	662(51.5)	
No	4234 (44.8)	733 (46.5)	623(48.5)	
Smoking during pregnancy (*n*, %)				< 0.001
Yes	2482(26.0)	338 (21.2)	300(23.1)	
No	7072 (74.0)	1256 (78.8)	996(76.9)	
Maternal antenatal anxiety symptoms (*n*, %)				0.225
Yes	1975 (23.1)	308 (21.1)	266(23.3)	
No	6578 (76.9)	1152 (78.9)	874(76.7)	
Maternal antenatal depressive symptoms (*n*, %)				0.007
Yes	1759 (20.0)	246(16.5)	235(20.1)	
No	7038 (80.0)	1241(83.5)	932(79.9)	
Child sex (*n*, %)				
Male	5143 (50.9)	918 (54.5)	726(52.8)	0.014
Female	4968 (49.1)	768 (45.6)	648(47.2)	
Gestational age at delivery (in weeks) (Mean/SD)	39.6(1.6)	39.8(1.8)	38.6(2.6)	< 0.001
Birth weight in kg (Mean/SD)	3.4(0.5)	3.4(0.5)	3.2(0.7)	< 0.001

*P* values correspond to Pearson’s chi-square test for categorical variables and one-way ANOVA for numerical variables; % refers to column percentages

*CSE* Certificate of Secondary Education

We also compared the characteristics of mothers who gave birth by elective CS to those with emergency CS. Compared with mothers who gave birth by elective CS, those who had emergency CS were younger at childbirth, never married, nulliparous, had hypertensive disorders during pregnancy, smoked tobacco, and were less likely to be overweight/obese (Table S1).

Table 2 shows the proportion of children being categorised as ‘borderline/abnormal’ for total behavioural difficulties and each SDQ sub-scale from age 3 to 16 years stratified by sex. Overall, more boys than girls had total behavioural difficulties, hyperactivity/inattention problems, conduct problems and peer relationship problems. In contrast, the proportion of children scoring in the ‘borderline/abnormal’ range for emotional problems was higher for girls than boys. However, no evidence of interaction between mode of delivery and gender was found at any age group (*P* ≥ 0.08 for interaction).

**Table 2:** Proportion of borderline/abnormal total and sub-scale SDQ scores by age and sex

Emotional and behavioural problems	Sex	3 years (*n* = 9788)	7 years (*n* = 8214)	9 years (*n* = 7877)	11 years (*n* = 7196)	16 years (*n* = 5502)
Total behavioural difficulties (*n*, %)	Total	3836 (39.1%)	884(10.8)	795(10.1)	701(9.7)	461 (8.4)
	Male	2122(41.9)	527(12.5)	475(11.9)	429(11.9)	205(7.7)
	Female	1714(36.3)	357(8.9)	320(8.2)	272(7.6)	256(8.9)
Chi-square (*χ*^2^) and *P* value		*χ*^2^ = 32.9; < 0.001	*χ*^2^ = 26.8; < 0.001	*χ*^2^ = 30.2; < 0.001	*χ*^2^ = 32.9; < 0.001	*χ*^2^ = 2.67; 0.102
Hyperactivity/inattention problems (*n*, %)	Total	708(7.2)	1517(18.5)	1060(13.4)	868(12.1)	544(9.8)
	Male	432(9.5)	997(23.6)	694(17.4)	595(16.6)	340(12.7)
	Female	276(5.8)	520(13.0)	366(9.4)	273(7.6)	204(7.1)
Chi-square (*χ*^2^) and *P* value		*χ*^2^ = 26.4; < 0.001	*χ*^2^ = 153.7; < 0.001	*χ*^2^ = 109.2; < 0.001	*χ*^2^ = 136.5; < 0.001	*χ*^2^ = 49.1; < 0.001
Conduct problems (*n*, %)	Total	6204(63.9)	1989(24.1)	1395(17.6)	1152(16.0)	685(12.4)
	Male	3342(66.8)	1091(25.8)	774(19.4)	635(17.7)	304(11.4)
	Female	2862(61.0)	898(22.4)	621(15.9)	517(14.3)	381(13.2)
Chi-square (*χ*^2^) and *P* value		*χ*^2^ = 35.2; < 0.001	*χ*^2^ = 12.9; < 0.001	*χ*^2^ = 16.8; < 0.001	*χ*^2^ = 14.9; < 0.001	*χ*^2^ = 4.4; 0.04
Emotional symptoms (*n*, %)	Total	2531(25.9)	1058(12.9)	1073(13.6)	899(12.5)	759(13.7)
	Male	1272(25.1)	500(11.8)	461(11.6)	397(11.1)	223(8.4)
	Female	1259(26.6)	558(13.9)	612(15.7)	502(14.0)	536(18.7)
Chi-square (*χ*^2^) and *P* value		*χ*^2^ = 2.9; 0.09	*χ*^2^ = 8.2; 0.004	*χ*^2^ = 28.0; < 0.001	*χ*^2^ = 13.6; < 0.001	*χ*^2^ = 124.8; < 0.001
Peer-relationship problems (*n*, %)	Total	*	1189(14.4)	1262(16.0)	1141(15.9)	866(15.7)
	Male	*	686(16.2)	712(17.8)	641(17.8)	457(17.1)
	Female	*	503(12.5)	550(14.1)	500(13.9)	409(14.3)
Chi-square (*χ*^2^) and *P* value			*χ*^2^ = 22.7; < 0.001	*χ*^2^ = 20.7; < 0.001	*χ*^2^ = 20.9; < 0.001	*χ*^2^ = 8.7; 0.003

Children were categorised as ‘borderline/abnormal’ if they scored in the ‘abnormal’ or ‘borderline’ ranges, according to cut-offs suggested by Goodman for total behavioural difficulties and for each SDQ sub-scale

Binomial cut-off: total difficulties (≥ 14), hyperactivity (≥ 6), conduct problems (≥ 3), emotional symptoms (≥ 4), peer relationship problems (≥ 3), pro-social behaviour (≤ 5)

^*^Not measured/no data available

Table S2 shows the mean scores for each SDQ sub-scale and total difficulties. The mean (standard deviation) score of total difficulties was 12.5 (5.7) at age 3 years and 6.2 (4.8) at age 16 years. While the mean SDQ scores for total difficulties and externalising behaviours (hyperactivity/inattention problems and conduct problems) were decreased as the child's age increased, internalising problems (emotional symptoms and pee-relationship problems) remained the same.

Univariable and multivariable associations between mode of delivery and offspring emotional and behavioural problems at five developmental time points (3, 7, 9, 11, and 16 years) are shown in Tables 3 and 4, respectively. In the univariable analysis, caesarean delivery was associated with peer relationship problems at the ages of 7 and 16 years. The unadjusted stratified analysis showed that emergency but not elective CS was associated with peer relationship problems at both ages. We also found an association between assisted (vacuum/forceps and/breech) vaginal delivery and emotional problems in children at ages 3, 7 and 11 years. After accounting for potential confounders, such as maternal depressive symptoms, hypertensive disorders during pregnancy and gestational diabetes, only the associations between elective caesarean delivery and peer relationship problems in offspring at age 7 years remained (OR = 1.43; 95% CI 1.01–2.04) (Table 4) and was further attenuated after we restricted our analysis to term babies (OR = 1.38; 95% CI 0.96–1.98) (Table S3).

**Table 3:** Association between mode of delivery and emotional and behavioural problems in children and adolescents (univariable analysis)

Offspring age	Mode of delivery	Children with SDQ ≥ 14	Unadjusted; OR (95% CI)					
			Total behavioural difficulties	Emotional symptoms	Peer-relationship problems	Hyperactivity/inattention problems	Conduct problems	Prosocial behaviours
3 years (*n* = 7074)	SVD (*n* = 5371)	2037	1	1	*	1	1	*
	AVD (*n* = 993)	407	1.14(0.99–1.30)	1.18(1.01–1.37)	*	0.90(0.69–1.17)	0.95(0.83–1.09)	*
	CS (*n* = 710)	276	1.02(0.87–1.20)	0.91(0.75–1.09)	*	1.00(0.74–1.35)	0.93(0.79–1.10)	*
	Elective CS (*n* = 272)	104	0.99(0.77–1.28)	0.82(0.61–1.10)	*	0.87(0.53–1.42)	0.93(0.73–1.20)	*
	Emergency CS (n = 439)	172	1.05(0.86–1.28)	0.97(0.77–1.22)	*	1.07(0.75–1.53)	0.94(0.77–1.15)	*
7 years (*n* = 6114)	SVD (*n* = 4621)	462	1	1	1	1	1	1
	AVD (*n* = 882)	85	0.96(0.75–1.22)	1.33(1.09–1.63)	1.02(0.82–1.26)	0.84(0.69–1.02)	1.04(0.88–1.24)	0.87(0.67–1.13)
	CS (*n* = 611)	68	1.15(0.88–1.50)	1.25(0.98–1.59)	1.42(1.14–1.78)	1.18(0.96–1.46)	1.06(0.87–1.29)	1.14(0.86–1.50)
	Elective CS (*n* = 237)	23	0.96(0.62–1.49)	1.17(0.80–1.71)	1.39(0.99–1.96)	1.19(0.86–1.64)	1.01(0.74–1.37)	1.42(0.96–2.09)
	Emergency CS (*n* = 374)	45	1.22(0.88–1.70)	1.31(0.98–1.77)	1.40(1.06–1.85)	1.14(0.88–1.49)	1.07(0.84–1.37)	0.92(0.64–1.34)
9 years (*n* = 5639)	SVD (*n* = 4272)	397	1	1	1	1	1	1
	AVD (*n* = 813)	68	0.89(0.68–1.17)	1.07(0.86–1.34)	1.03(0.84–1.28)	0.89(0.70–1.12)	1.00(0.82–1.23)	0.79(0.58–1.07)
	CS (*n* = 554)	50	0.97(0.71–1.32)	1.12(0.87–1.45)	1.20(0.94–1.52)	1.00(0.77–1.30)	0.91(0.72–1.17)	0.95(0.67–1.32)
	Elective CS (*n* = 207)	17	0.86(0.52–1.43)	1.26(0.86–1.86)	1.18(0.81–1.71)	0.85(0.55–1.33)	1.00(0.69–1.45)	1.11(0.68–1.83)
	Emergency CS (*n* = 347)	33	1.02(0.70–1.48)	1.05(0.76–1.45)	1.19(0.89–1.59)	1.07(0.78–1.47)	0.89(0.66–1.21)	0.87(0.57–1.34)
11 years (*n* = 5211)	SVD (*n* = 3909)	346	1	1	1	1	1	1
	AVD (*n* = 776)	75	1.10(0.85–1.43)	1.31(1.05–1.64)	1.18(0.96–1.45)	1.06(0.84–1.35)	0.96(0.77–1.19)	1.05(0.78–1.41)
	CS (*n* = 526)	52	1.13(0.83–1.54)	1.23(0.94–1.61)	1.22(0.96–1.56)	1.02(0.76–1.35)	0.88(0.67–1.14)	1.09(0.77–1.54)
	Elective CS (*n* = 190)	14	0.81(0.46–1.40)	1.40(0.93–2.10)	0.84(0.54–1.30)	0.97(0.61–1.53)	0.72(0.46–1.14)	1.28(0.77–2.14)
	Emergency CS (*n* = 336)	38	1.30(0.91–1.85)	1.15(0.82–1.60)	1.43(1.07–1.89)	1.02(0.72–1.45)	0.95(0.69–1.30)	0.96(0.62–1.50)
16 years (*n* = 4071)	SVD (*n* = 3021)	232	1	1	1	1	1	1
	AVD (*n* = 644)	47	0.95(0.68–1.31)	0.95(0.74–1.22)	1.15(0.92–1.46)	0.93(0.69–1.25)	1.10(0.85–1.42)	1.19(0.92–1.53)
	CS (*n* = 406)	38	1.24(0.87–1.78)	1.01(0.75–1.37)	1.31(1.00–1.72)	1.07(0.76–1.50)	0.91(0.65–1.27)	0.80(0.56–1.13)
	Elective CS (*n* = 136)	7	0.64(0.30–1.39)	0.83(0.49–1.41)	0.72(0.42–1.24)	0.72(0.38–139)	0.97(0.57–1.66)	0.41(0.19–0.88)
	Emergency CS (*n* = 270)	31	1.55(1.04–2.30)	1.09(0.77–1.55)	1.63(1.20–2.21)	1.24(0.84–1.82)	0.86(0.57–1.29)	1.04(0.70–1.52)

*SVD* Spontaneous vaginal delivery, *AVD* assisted vaginal delivery, *CS* Caesarean section

^*^Not measured/no data available, 1 reference category

**Table 4:** Association between mode of delivery and emotional and behavioural problems in children and adolescents (multivariate model)

Offspring age	Mode of delivery	Children with SDQ ≥ 14	Adjusted; OR (95% CI)#					
			Total behavioural difficulties	Emotional symptoms	Peer-relationship problems	Hyperactivity/inattention problems	Conduct problems	Prosocial behaviours
3 years (*n* = 7074)	SVD (*n* = 5371)	2037	1	1	*	1	1	*
	AVD (*n* = 993)	407	1.12(0.96–1.30)	1.01(0.86–1.18)	*	0.84(0.64–1.12)	1.10(0.95–1.28)	*
	CS (*n* = 710)	276	1.03(0.87–1.22)	0.87(0.72–1.06)	*	0.99(0.73–1.35)	1.00(0.85–1.19)	*
	Elective CS (*n* = 272)	104	1.04(0.80–1.34)	0.89(0.66–1.21)	*	0.88(0.53–1.44)	0.91(0.70–1.18)	*
	Emergency CS (*n* = 439)	172	1.04(0.84–1.28)	0.86(0.69–1.09)	*	1.05(0.72–1.53)	1.09(0.88–1.34)	*
7 years (*n* = 6114)	SVD (*n* = 4621)	462	1	1	1	1	1	1
	AVD (*n* = 882)	85	0.93(0.72–1.21)	1.25(1.01–1.56)	0.95(0.76–1.20)	0.83(0.67–1.03)	1.14(0.95–1.37)	0.90(0.69–1.19)
	CS (*n* = 611)	68	1.11(0.83–1.47)	1.27(0.99–1.63)	1.36(1.08–1.72)	1.17(0.93–1.46)	1.10(0.90–1.35)	1.12(0.84–1.49)
	Elective CS (*n* = 237)	23	0.98(0.62–1.55)	1.26(0.85–1.85)	1.43(1.01–2.04)	1.22(0.87–1.70)	1.03(0.75–1.41)	1.32(0.89–1.97)
	Emergency CS (*n* = 374)	45	1.15(0.81–1.63)	1.29(0.95–1.76)	1.27(0.95–1.71)	1.11(0.83–1.47)	1.15(0.89–1.48)	0.94(0.64–1.38)
9 years (*n* = 5639)	SVD (*n* = 4272)	397	1	1	1	1	1	1
	AVD (*n* = 813)	68	0.89(0.67–1.19)	1.09(0.86–1.38)	0.96(0.77–1.20)	0.88(0.68–1.13)	1.06(0.85–1.31)	0.74(0.54–1.02)
	CS (*n* = 554)	50	0.96(0.70–1.33)	1.11(0.85–1.45)	1.12(0.88–1.43)	0.96(0.73–1.27)	0.94(0.72–1.21)	0.91(0.64–1.29)
	Elective CS (*n* = 207)	17	0.90(0.54–1.51)	1.29(0.86–1.91)	1.19(0.82–1.74)	0.88(0.56–1.39)	1.02(0.69–1.51)	1.15(0.70–1.92)
	Emergency CS (*n* = 347)	33	0.99(0.67–1.47)	1.02(0.73–1.43)	1.06(0.78–1.44)	1.00(0.71–1.40)	0.91(0.66–1.26)	0.80(0.51–1.25)
11 years (*n* = 5211)	SVD (*n* = 3909)	346	1	1	1	1	1	1
	AVD (*n* = 776)	75	1.20(0.90–1.60)	1.42(1.11–1.81)	1.08(0.86–1.35)	1.11(0.86–1.15)	0.94(0.74–1.19)	0.95(0.69–1.31)
	CS (*n* = 526)	52	1.10(0.80–1.52)	1.23(0.93–1.63)	1.11(0.86–1.43)	0.99(0.74–1.34)	0.86(0.65–1.13)	1.07(0.75–1.54)
	Elective CS (*n* = 190)	14	0.80(0.45–1.40)	1.39(0.91–2.10)	0.82(0.53–1.28)	0.97(0.60–1.56)	0.75(0.47–1.19)	1.36(0.57–1.44)
	Emergency CS (*n* = 336)	38	1.28(0.87–1.87)	1.14(0.80–1.63)	1.26(0.93–1.69)	1.00(0.69–1.44)	0.91(0.65–1.27)	0.91(0.57–1.44)
16 years (*n* = 4071)	SVD (*n* = 3021)	232	1	1	1	1	1	1
	AVD (*n* = 644)	47	0.87(0.61–1.23)	0.94(0.71–1.23)	0.99(0.77–1.27)	0.95(0.69–1.30)	1.05(0.80–1.38)	1.09(0.83–1.43)
	CS (*n* = 406)	38	1.21(0.83–1.77)	0.99(0.72–1.36)	1.14(0.86–1.51)	1.12(0.78–1.61)	0.90(0.64–1.27)	0.79(0.55–1.13)
	Elective CS (*n* = 136)	7	0.65(0.30–1.44)	0.83(0.48–1.43)	0.70(0.50–1.21)	0.74(0.38–1.45)	1.01(0.59–1.74)	0.45(0.21–0.98)
	Emergency CS (*n* = 270)	31	1.50(0.98–2.29)	1.06(0.73–1.55)	1.35(0.98–1.87)	1.34(0.88–2.02)	0.83(0.54–1.27)	0.98(0.65–1.47)

*SVD* spontaneous vaginal delivery, *AVD* assisted vaginal delivery, *CS* caesarean section

^#^Adjusted for maternal age, educational status, ethnicity, parity, pre-pregnancy body mass index, pregnancy diabetes, infection during pregnancy, hypertensive disorders during pregnancy, alcohol consumption during pregnancy, smoking during pregnancy, maternal antenatal depression and anxiety and offspring sex and gestational age at delivery

^*^Not measured/no data available

Most associations between assisted vaginal delivery and emotional problems were no longer seen after adjustment except with emotional problems at the age of 11 years, where we found that children of mothers who gave birth by assisted vaginal delivery were 42% more likely to have emotional problems (OR = 1.42; 95% CI 1.11–1.81). Limiting our analysis to term babies did not alter our results (Table S3). Further stratified analyses by types of assisted delivery showed that vacuum delivery (OR = 1.43; 95% CI 1.01–2.02) and assisted breech delivery (OR = 1.92; 95% CI 1.01–3.67) were found to be associated with emotional problems. The association between forceps delivery and emotional problems was approach statistically significant (OR = 1.37; 95% CI 0.97–1.93, *P* = 0.07). Results were broadly consistent when we repeated the analyses using the imputed datasets (Table S4).

## Discussion

In this large longitudinal study, we investigated the association between mode of delivery and offspring emotional and behavioural problems at five developmental time periods (at ages 3, 7, 9, 11, and 16). After accounting for a range of potential confounders, we found no evidence of an association between CS delivery modes (elective or emergency) and emotional and behavioural measures in offspring (total difficulties, conduct problems, hyperactivity/inattention problems, peer relationship problems, emotional problems and problems with pro-social behaviours) across all developmental time periods. Our findings provide reassurance to those mothers concerned about the potential risks of birth by CS on the mental health of their children at later age. We also found no evidence of associations between assisted vaginal delivery and emotional and behavioural problems at all ages except at age 11, where assisted vaginal delivery was associated with a 42% increased risk of emotional problems.

Our findings are broadly consistent with few existing studies that have reported no significant association between mode of delivery and child behavioural problems [31–34]. In the UK Millennium cohort study, Curran et al. [31] found no association between modes of delivery and behavioural difficulties (also measured by SDQ) in children aged 7 years. Similarly, a longitudinal study conducted in Ireland [32] and a birth cohort study in China [33] reported no association between CS and abnormal child behaviour. Our findings partially agreed with Rutayisire et al.’s [30] study, which has reported mixed results. In Rutayisire et al.’s study, while CS was associated with abnormal total SDQ score and problems with pro-social behaviour in this study, no significant association was found between birth by CS and the risk of having conduct problems, hyperactivity/inattention problems, peer relationship problems, and emotional problems [29].

Our findings are in contrast with studies that reported associations [28, 29]. A retrospective cohort study conducted in China found that children born by assisted vaginal delivery were more likely to have externalising and internalising problems than those delivered by spontaneous vaginal delivery, whereas children born by CS were less likely to have externalising scores [29]. A Russian study by Kelmanson [28] also found associations between CS delivery and internalising problems in 5-year-old children. The discrepancy could be attributed to the tool used to measure emotional and behavioural problems, age at outcome assessment, study design, sample size, and level of adjustment for confounding. While previous studies examined the association in a sample of children aged 4–6 years [28, 29], we examined the associations at five developmental ages, from 3 to 16 years. In addition, Kelmanson's [28] study is a small case–control study (*n* = 80), and both studies [28, 29] did not adjust for important confounders, such as parity, perinatal maternal mental health, perinatal smoking and alcohol use, which may have led to overestimation of the risk observed in these studies. In the current study, we used data from a large birth cohort study (*n* = 4071 +) and were able to comprehensively assess confounders in our analysis. Finally, while previous studies used Child Behaviour Checklist (CBCL) to assess children’s emotional and behavioural problems [28, 29], we used the SDQ. Although both measures are valid and reliable instruments for assessing child emotional and behavioural problems [39, 45], the SDQ is much shorter and has better specificity, whereas the CBCL has a higher sensitivity [46].

We found a small increased risk of emotional problems at age 11 in children born via assisted vaginal delivery. The observed association between assisted vaginal delivery and emotional problems is possibly related to cortisol response. Existing evidence suggested that children born by assisted vaginal delivery experienced the highest levels of stress during birth compared to those born by spontaneous vaginal delivery [47–49], and CS and high cortisol response was associated with an increased risk of childhood and adolescent psychopathology, including emotional difficulties [50, 51]. However, the existing evidence is mixed with respect to this association which raises the possibility that ours may be a chance finding. Consistent with our finding, Li et al. [29] study also reported a 41% (OR = 1.41; 95% CI 1.08–1.84) increased risk of internalising problem scores in children born by assisted vaginal delivery. It is also important to note that Li et al. [29] only looked 4–6-year-old children born from primiparous women. This study also used CBCL to measure children internalising problems and did not report findings from the CBCL sub-scales. However, more comparable studies by Maher et al. [34] and Leung et al. [33] found no evidence of associations between assisted vaginal delivery and emotional problems at age 11 when measured by SDQ. Taken together, the overall evidence signals the need for further research, particularly in older children, to confirm the robustness of our findings as well as to better understand the relationship between assisted vaginal delivery and offspring emotional development.

### Strength and limitations

This study has considerable strengths. The prospective design, large sample size, long-term follow-up period and the availability of data on a wide range of known confounders are some of the strengths of this study. The population-based samples limit the possibility of selection bias. We were able to classify CS into planned or emergency and vaginal delivery into assisted and spontaneous vaginal delivery, which was a limitation of most existing studies [28, 30, 33]. In addition, measures of offspring emotional and behavioural wellbeing at several time points allowed us to draw conclusions across the child’s development rather than being restricted to an individual time period.

The following limitations should also be considered. Children’s emotional and behavioural problems were reported by parents. Reliance on self-reports may lead to misclassification bias. As parents may underestimate behavioural problems, and any such misclassification would tend to weaken the association. We also relied on symptoms rather than diagnostic instruments. This may lead to random measurement errors. However, SDQ is a valid and reliable tool for assessing emotional and behavioural problems in offspring and has been widely used in research and clinical practice [39, 40]. While our data allowed us to adjust for a wide range of confounding factors, we cannot exclude the possibility of residual confounding. Attrition may also compromise the generalizability of the findings and may potentially bias estimates. Children with behavioural disorders are more likely to be lost to follow-up, which may bias our results towards the null. However, multiple imputations were used to address the limitation of missing data and reanalysis following data imputation indicated that our results were robust. In addition, previous work in ALSPAC has suggested that selective drop-out does not bias the prediction of the risk of behavioural disorders [52]. Consistent with this, other research has also showed that loss to follow-up rarely affects estimates of associations [53], suggesting attrition due to missing data was unlikely to have biased our results.

### Implications

We found no evidence of associations between elective or emergency CS and behavioural outcomes in children and adolescents. That said, CS is a surgical procedure with potential short- and long-term health risks. As such, it should ideally only be undertaken when medically necessary. With the exception of emotional problems at age 11, all the observed associations between obstetric mode of delivery and emotional and peer relationship problems in children did not remain after adjustment and/or stratified analysis, suggesting associations were likely due to confounding.

## Conclusion

We found small evidence of associations between assisted vaginal deliveries and increased risk of emotional problems at age 11. No evidence of associations between CS delivery and emotional and behavioural problems was observed in offspring at all ages. Further research is needed to confirm our findings.

## Supplementary Information

Below is the link to the electronic supplementary material.

Supplementary file1 (DOCX 24 KB)

Supplementary file2 (DOCX 20 KB)

Supplementary file3 (DOCX 25 KB)

Supplementary file4 (DOCX 22 KB)

## Data Availability

Access to data and supporting documentation can be requested from the ALSPAC (http://www.bristol.ac.uk/alspac/researchers/access/).
