# Supplementary material for: Association between obstetric mode of delivery and emotional and behavioural problems in children and adolescents: the children of the 90s health study
Source: Soc Psychiatry Psychiatr Epidemiol. 2022 Oct 14;58(6):949–60. doi: 10.1007/s00127-022-02374-z (PMC10241698; doi:10.1007/s00127-022-02374-z)
Supplement: Supplementary file 1 — Supplementary file1 (DOCX 24 KB) [file 127_2022_2374_MOESM1_ESM.docx]

Table S1. Characteristics of study who gave birth by caesarean section

| Characteristics | Caesarean section (*n* = 1397) | | |
| --- | --- | --- | --- |
|  | Elective  (*n* = 492) | Emergency (*n* = 905) | *P-value* |
| Maternal age at delivery (mean, SD) | 29.6 (4.7) | 28.1 (5.1) | < 0.001 |
| Maternal education (%) |  |  | 0.382 |
| CSE | 14.4 | 15.5 |  |
| Vocational | 8.4 | 10.9 |  |
| O level | 35.0 | 36.1 |  |
| A level | 29.0 | 24.3 |  |
| Degree | 13.2 | 13.2 |  |
| Marital status (%) |  |  | < 0.001 |
| Married | 82.4 | 73.9 |  |
| Widowed/divorced/Separated | 8.1 | 5.6 |  |
| Never married | 9.5 | 20.5 |  |
| Parity ( %) |  |  | < 0.001 |
| Nullipara | 27.3 | 67.6 |  |
| Multipara | 72.7 | 32.4 |  |
| Pre-pregnancy BMI (Kg/m^2^) (%) |  |  | 0.047 |
| <18.5 | 4.7 | 3.5 |  |
| 18.5 -24.99 | 61.3 | 68.4 |  |
| ≥25 | 34.0 | 28.1 |  |
| Hypertensive disorders during pregnancy (%) |  |  | < 0.001 |
| Yes | 19.9 | 33.9 |  |
| No | 80.1 | 66.1) |  |
| Pregnancy diabetes status (%) |  |  | 0.102 |
| Glycosuria or diabetes (existing /gestational) | 8.5 | 6.1 |  |
| No glycosuria or diabetes | 91.5 | 93.9 |  |
| Any infection during pregnancy ( %) |  |  | 0.043 |
| Yes | 26.6 | 21.5 |  |
| No | 73.4 | 78.5 |  |
| Alcohol drinking in pregnancy (%) |  |  | 0.213 |
| Yes | 53.8 | 49.8 |  |
| No | 46.2 | 50.2 |  |
| Smoking during pregnancy (%) |  |  | 0.029 |
| Yes | 19.7 | 25.0 |  |
| No | 80.3 | 75.0 |  |
| Maternal antenatal anxiety symptoms ( %) |  |  | 0.411 |
| Yes | 24.6 | 22.7 |  |
| No | 75.4 | 77.5 |  |
| Maternal antenatal depressive symptoms ( %) |  |  | 0.782 |
| Yes | 20.5 | 19.8 |  |
| No | 79.5 | 80.2 |  |
| Child sex (%) |  |  | 0.144 |
| Male | 50.2 | 54.3 |  |
| Female | 49.8 | 45.7 |  |
| Gestational age at delivery (in weeks) (Mean/SD) | 38.4 (1.5) | 38.7 (3.0) | 0.026 |
| Birth weight in kg (Mean/SD) | 3.3(0.5) | 3.2(0.8) | 0.005 |
| Certificate of Secondary Education (CSE), P-values correspond to Pearson's chi-square test for categorical variables and one-way ANOVA for numerical variables; % refers to column percentages. | | | |
