# Supplementary material for: Association between obstetric mode of delivery and emotional and behavioural problems in children and adolescents: the children of the 90s health study
Source: Soc Psychiatry Psychiatr Epidemiol. 2022 Oct 14;58(6):949–60. doi: 10.1007/s00127-022-02374-z (PMC10241698; doi:10.1007/s00127-022-02374-z)
Supplement: Supplementary file 2 — Supplementary file2 (DOCX 20 KB) [file 127_2022_2374_MOESM2_ESM.docx]

Table S2. The mean scores of total behavioural difficulties and SDQ sub-scales.

| Emotional and behavioural problems | Mean (s.d.) | | | | |
| --- | --- | --- | --- | --- | --- |
|  | 3 years | 7 years | 10 years | 13 years | 16 years |
| Total difficulties | 12.5(5.7) | 7.5(4.8) | 6.9(5.0) | 6.6(5.0) | 6.2(4.8) |
| Emotional symptoms | 2.6(1.7) | 1.5(1.7) | 1.5(1.8) | 1.5(1.7) | 1.5(1.9) |
| Peer relationship problems | --- | 1.1(1.4) | 1.1(1.5) | 1.1(1.6) | 1.1(1.5) |
| Hyperactivity/ inattention | 2.6(1.81) | 3.4(2.4) | 3.0(2.3) | 2.8(2.2) | 2.6(2.1) |
| Conduct problems | 3.5(2.2) | 1.6(1.5) | 1.3(1.4) | 1.2(1.4) | 1.0(1.4) |
| Prosocial behaviour | --- | 8.2(1.8) | 8.3(1.7) | 8.3(1.7) | 8.0(1.9) |
| S.d. = Standard deviation. --- Not measured/no data available  Total difficulties score range: 0–40; other domains score range: 0–10. Higher scores represent higher problems except for pro-social behaviour, where lower scores represent greater difficulties. | | | | | |
