# Supplementary material for: Association between obstetric mode of delivery and emotional and behavioural problems in children and adolescents: the children of the 90s health study
Source: Soc Psychiatry Psychiatr Epidemiol. 2022 Oct 14;58(6):949–60. doi: 10.1007/s00127-022-02374-z (PMC10241698; doi:10.1007/s00127-022-02374-z)
Supplement: Supplementary file 3 — Supplementary file3 (DOCX 25 KB) [file 127_2022_2374_MOESM3_ESM.docx]

Table S3. Association between mode of delivery and emotional and behavioural problems in children and adolescents: Restricted to term berth

| Offspring age | Mode of delivery | Adjusted; OR (95% CI) | | | | |
| --- | --- | --- | --- | --- | --- | --- |
|  |  | Emotional symptoms | Peer-relationship problems | Hyperactivity/ inattention problems | Conduct problems | Prosocial behaviours |
| 3 years | Spontaneous vaginal delivery | 1 | * | 1 | 1 | * |
|  | Assisted vaginal delivery | 1.03(0.87-1.21) | * | 0.82(0.62-1.10) | 1.09(0.94-1.28) | * |
|  | Caesarean section | 0.85(0.69-1.04) | * | 1.04(0.76-1.42) | 1.02(0.86-1.22) | * |
|  | Elective CS | 0.89(0.65-1.21) | * | 0.90(0.55-1.48) | 0.95(0.73-1.23) | * |
|  | Emergency CS | 0.84(0.65-1.08) | * | 1.13(0.77-1.66) | 1.10(0.88-1.37) | * |
| 7 years | Spontaneous vaginal delivery | 1 | 1 | 1 | 1 | 1 |
|  | Assisted vaginal delivery | 1.25(1.00-1.56) | 0.93(0.74-1.17) | 0.82(0.66-1.02) | 1.15(0.96-1.39) | 0.86(0.65-1.14) |
|  | Caesarean section | 1.24(0.95-1.60) | 1.29(1.01-1.64) | 1.15(0.91-1.45) | 1.11(0.89-1.37) | 1.07(0.80-1.45) |
|  | Elective CS | 1.14 (0.76-1.70) | 1.38(0.96-1.98) | 1.10(0.78-1.56) | 1.04(0.76-1.43) | 1.24(0.82-1.87) |
|  | Emergency CS | 1.32(0.96-1.82) | 1.18(0.86-1.62) | 1.15(0.85-1.55) | 1.15(0.88-1.51) | 0.89(0.59-1.36) |
| 9 years | Spontaneous vaginal delivery | 1 | 1 | 1 | 1 | 1 |
|  | Assisted vaginal delivery | 1.06(1.83-1.36) | 0.99(0.79-1.24) | 0.89(0.69-1.15) | 1.09(0.87-1.36) | 0.78(0.56-1.07) |
|  | Caesarean section | 1.14(0.86-1.50) | 1.18(0.92-1.53) | 0.96(0.72-1.29) | 0.94(0.72-1.22) | 0.89(0.62-1.30) |
|  | Elective CS | 1.26(0.85-1.89) | 1.15(0.78-1.70) | 0.86(0.54-1.36) | 1.02(0.69-1.50) | 1.20(0.72-1.99) |
|  | Emergency CS | 1.06(0.74-1.51) | 1.19(0.86-1.64) | 1.02(0.71-1.47) | 0.92(0.65-1.29) | 0.73(0.44-1.22) |
| 11 years | Spontaneous vaginal delivery | 1 | 1 | 1 | 1 | 1 |
|  | Assisted vaginal delivery | 1.44(1.12-1.85) | 1.06(0.85-1.34) | 1.18(0.90-1.54) | 0.92(0.72-1.17) | 0.95(0.68-1.31) |
|  | Caesarean section | 1.21(0.90-1.62) | 1.02(0.78-1.34) | 0.97(0.70-1.34) | 0.86(0.64-1.15) | 1.08(0.74-1.56 |
|  | Elective CS | 1.31(0.85-2.02) | 0.76(0.48-1.20) | 0.94(0.58-1.53) | 0.72(0.45-1.15 | 1.29(0.75-2.21) |
|  | Emergency CS | 1.14(0.79-1.67) | 1.18(0.86-1.63) | 0.97(0.65-1.46) | 0.93(0.65-1.33) | 0.93(0.57-1.51) |
| 16 years | Spontaneous vaginal delivery | 1 | 1 | 1 | 1 | 1 |
|  | Assisted vaginal delivery | 0.93(0.71-1.23) | 1.01(0.79-1.30) | 0.98(0.71-1.34) | 1.05(0.79-1.39) | 1.11(0.84-1.46) |
|  | Caesarean section | 0.95(0.67-1.33) | 1.13(0.84-1.52) | 1.16(0.80-1.68) | 0.91(0.64-1.30) | 0.73(0.49-1.08) |
|  | Elective CS | 0.74(0.42-1.31) | 0.72(0.42-1.26) | 0.76(0.39-1.49) | 1.03(0.59-1.78) | 0.46(0.21-0.99) |
|  | Emergency CS | 1.06(0.71-1.58) | 1.36(0.96-1.91) | 1.40(0.91-2.17) | 0.83(0.53-1.29) | 0.92(0.59-1.43) |
| Adjusted for maternal age, educational status, ethnicity, parity, pre-pregnancy body mass index, pregnancy diabetes, infection during pregnancy, hypertensive disorders during pregnancy, alcohol consumption during pregnancy, smoking during pregnancy, maternal antenatal depression and anxiety and offspring sex and gestational age at delivery.  * Not measured/no data available | | | | | | |
