# Supplementary material for: Association between obstetric mode of delivery and emotional and behavioural problems in children and adolescents: the children of the 90s health study
Source: Soc Psychiatry Psychiatr Epidemiol. 2022 Oct 14;58(6):949–60. doi: 10.1007/s00127-022-02374-z (PMC10241698; doi:10.1007/s00127-022-02374-z)
Supplement: Supplementary file 4 — Supplementary file4 (DOCX 22 KB) [file 127_2022_2374_MOESM4_ESM.docx]

Table S4. Association between mode of delivery and emotional and behavioural problems in children and adolescents: using imputed dataset (*n* = 13171)

| Offspring age | Mode of delivery | Adjusted; OR (95% CI) | | | | |
| --- | --- | --- | --- | --- | --- | --- |
|  |  | Total behavioural difficulties | Emotional symptoms | Peer-relationship problems | Hyperactivity/ inattention problems | Conduct problems |
| 3 years | Spontaneous vaginal delivery | 1 | 1 | * | 1 | 1 |
|  | Assisted vaginal delivery | 1.10(0.97-1.25) | 1.02(0.89-1.17) | * | 0.85(0.66-1.09) | 1.11(0.98-1.26) |
|  | Caesarean section | 0.98(0.85-1.12) | 0.87(0.74-1.02) | * | 0.98(0.76-1.26) | 1.01(0.88-1.16) |
| 7 years | Spontaneous vaginal delivery | 1 | 1 | 1 | 1 | 1 |
|  | Assisted vaginal delivery | 0.97(0.79-1.19) | 1.17(0.98-1.39) | 0.99(0.84-1.19) | 0.94(0.79-1.12) | 1.07(0.92-1.24) |
|  | Caesarean section | 1.07(0.86-1.32) | 1.08(0.89-1.33) | 1.15(0.96-1.37) | 1.09(0.92-1.30) | 1.01(0.86-1.19) |
| 9 years | Spontaneous vaginal delivery | 1 | 1 | 1 | 1 | 1 |
|  | Assisted vaginal delivery | 1.09(0.89-1.35) | 1.14(0.95-1.37) | 0.99(0.83-1.19) | 096(0.79-1.16) | 1.05(0.88-1.24) |
|  | Caesarean section | 0.99(0.77-1.27) | 1.05(0.86-1.28) | 1.11(0.92-1.32) | 1.01(0.82-1.25) | 0.99(0.83-1.17) |
| 11 years | Spontaneous vaginal delivery | 1 | 1 | 1 | 1 | 1 |
|  | Assisted vaginal delivery | 0.91(0.71-1.17) | 1.19(1.97-1.47) | 1.03(0.86-1.23) | 1.06(0.87-1.28) | 0.96(0.79-1.15) |
|  | Caesarean section | 1.03(0.79-1.34) | 1.16(0.95-1.42) | 1.09(0.90-1.31) | 1.02(0.81-1.26) | 0.98(0.81-1.20) |
| 16 years | Spontaneous vaginal delivery | 1 | 1 | 1 | 1 | 1 |
|  | Assisted vaginal delivery | 1.02(0.89-1.17) | 0.99(0.82-1.22) | 1.01(0.84-1.22) | 0.96(0.77-1.19) | 0.98(0.86-1.20) |
|  | Caesarean section | 0.87(0.74-1.02) | 0.99(0.80-1.24) | 1.09(0.89-1.34) | 1.03(0.82-1.31) | 0.97(0.78-1.21) |
| Adjusted for maternal age, educational status, ethnicity, parity, pre-pregnancy body mass index, pregnancy diabetes, infection during pregnancy, hypertensive disorders during pregnancy, alcohol consumption during pregnancy, smoking during pregnancy, maternal antenatal depression and anxiety and offspring sex and gestational age at delivery.  * Not measured/no data available | | | | | | |
